# Prevalence and factors associated with HIV and syphilis infection among children aged 0–36 months in Kilimanjaro, Tanzania: a community-based cross-sectional study

**DOI:** 10.1186/s41182-019-0183-4

**Published:** 2019-11-21

**Authors:** Nikolas A. S. Chotta, Melina Mgongo, Sia E. Msuya, Balthazar M. Nyombi, Jacqueline G. Uriyo, Babill Stray-Pedersen, Arne Stray-Pedersen

**Affiliations:** 1 Institute of Clinical Medicine, University of Oslo, Oslo, Norway; 2 Better Health for African Mothers and Children, P.O. Box 8418, Moshi, Tanzania; 3 Department of Community Health, Institute of Public Health, Kilimanjaro Christian Medical University College (KCMUCo), Moshi, Tanzania; 4 Department of Epidemiology and Biostatistics, Institute of Public Health, Kilimanjaro Christian Medical University College (KCMUCo), Moshi, Tanzania; 5 Department of Health Laboratory Sciences, Kilimanjaro Christian Medical University College (KCMUCo), Moshi, Tanzania; 6 Division of Women, Oslo University Hospital, Rikshospitalet, Oslo, Norway; 7 Department of Forensic Medicine, Institute of Public Health, University of Oslo, Oslo, Norway

**Keywords:** HIV, Syphilis, Infants, Children, Dual elimination, Mother-to-child transmission, PMTCT, Tanzania

## Abstract

**Background:**

Childhood mortality is high in sub-Saharan Africa. Mother-to-child transmission (MTCT) of HIV and congenital syphilis are among significant causes. Dual elimination of these two infections is one of the international goals. Community-based studies on the burden of HIV and syphilis among children will contribute to fine-tuning the interventions to achieve the elimination goal. This study aims to describe the prevalence of HIV and syphilis among children aged 0–36 months and associated factors in northern Tanzania.

**Methods:**

This was a community-based cross-sectional study, which was conducted in all the seven districts of Kilimanjaro region. Multistage sampling was used, and a total of 2452 children aged 0 to 36 months and their primary caretakers were enrolled. Interviews were conducted with the mother/caretaker, and dried blood samples were collected from the children and processed for laboratory diagnosis of HIV and syphilis. HIV ELISA was first performed on all the samples. Positive samples of children < 18 months were confirmed using PCR.

**Results:**

The prevalence of HIV among 2452 children aged 0–36 months was 1.7% (*n* = 42). There was a significant difference in the distribution of HIV by age of the child, maternal antenatal attendance, and breastfeeding history.

The prevalence of syphilis was 0.4% (*n* = 10). Five of the children were more than 1 year old. All children with a positive test for syphilis were from Moshi rural district, and their mothers consumed alcohol. No child was co-infected with HIV and syphilis.

**Conclusions:**

Though the prevalence of the two infections was low, detecting syphilis in children suggests a missed opportunity in screening women during pregnancy. The region may be on track with the goal to achieve dual elimination of mother-to-child transmitted HIV and syphilis. However, efforts are needed to reduce missed opportunities for screening women for syphilis and HIV early in pregnancy and retesting at 3rd trimester/delivery. Strategies to improve testing for HIV-exposed children are needed.

## Background

The under-five mortality rate (U5MR) has declined by 58% globally. It has declined from 93 deaths/1000 live births in 1990 to 39 deaths/1000 live births by the year 2017 [1]. The decline was uneven, with sub-Saharan Africa still experiencing high child mortality rates, at 74 deaths per 1000 live births in 2017 [1, 2]. The lessons learnt from the efforts to achieve the MDGs have created new opportunities for the Sustainable Development Goals (SDGs). The proposed targets of ending preventable neonatal and under-five child death are to reach a neonatal mortality rate of 12/1000 live births and under-five mortality rate below 25/1000 live births by the year 2030 [1–3].

Tanzania, a country in SSA, also recorded a decline in under-five and neonatal mortality rates. The U5MR declined from 166 per 1000 live births in 1990 to 54 per 1000 live births in 2017. Neonatal mortality declined from 38 per 1000 live births in 1990 to 21 per 1000 live births in 2017 [1]. The mortality rates are still unacceptably high and far from the 2030 targets. Preventable infections (like HIV and syphilis) are still significant contributors to these trends [1, 3]. AIDS still contributes to substantial mortality of children in SSA and in Tanzania. In Tanzania, AIDS is the fifth leading cause of death of children aged 1–59 months causing 5.7% of all deaths while it contributes to 3.9% of the deaths among children aged 1–59 months in SSA [1].

HIV and syphilis prevalence is still high among pregnant women in SSA [4–10]. Currently it is estimated that globally 1.4 million pregnant women are living with HIV, with 1.8 million children with HIV. More than 85% of the infections in children occur in SSA [6]. The World Health Organization (WHO) also estimated that globally every year about 1.86 million pregnant women have syphilis. The prevalence of syphilis among pregnant women in SSA is high, ranging between 2.5 and 17% [7, 8, 10–12].

Policies and programs for testing and treating both HIV and syphilis infections among pregnant women have been available for many years in SSA countries [5, 9, 10]. Thus, control and elimination of mother-to-child transmission (MTCT) of HIV and syphilis are possible. However, programmatic bottlenecks like poor coverage of ANC screening, especially for syphilis [5, 11–16], poor treatment of infected pregnant women [5, 17, 18], poor testing of exposed children [19, 20], and high loss to follow-up of mother-infant pairs have led to continued transmission of these infections to children [5, 21, 22]. SSA therefore has high rates of MTCT of HIV, the highest number of congenital syphilis cases, and more than 70% of 1.8 million children with HIV [5, 10, 12, 19, 21]. Apart from transmission, untreated HIV and syphilis in pregnancy are both associated with negative pregnancy outcomes (stillbirth, premature births, low birth weight and congenital infection for syphilis) [10, 11, 22–24].

The global community (WHO, UNAIDS, and other UN bodies) is calling for dual elimination of MTCT of HIV and syphilis [5, 9, 10]. Given the similar mode of transmission, coupled with high antenatal attendance, availability of point of care rapid diagnostic tests for both infections, and availability of treatment and care for both infections along the continuum of care provided in the same platform (RCH Clinics), it should be possible to achieve the elimination goal before 2030 [5, 9, 10, 13, 19, 25]. Some countries, like Cuba, had achieved dual elimination of MTCT of HIV and syphilis by 2015 [26].

Data on the effectiveness of prevention of mother-to-child transmission (PMTCT) programs for HIV and syphilis is needed to guide effective policy making at the national and local level [9, 10]. In Tanzania, the PMTCT of HIV program started in 2004, while antenatal syphilis screening and treatment were already in place. While HIV testing during pregnancy and ARV prophylaxis coverage are high (99% and 90% respectively), data for syphilis shows only 30–62% of pregnant women are screened for syphilis in Tanzania [11, 13, 16, 19, 22]. Data on congenital syphilis cases is limited, and only 55% of the HIV-exposed children are tested at 6–8 weeks (EID) in the country and fewer are tested at 18 months [19]. Thus, data on the transmission of MTCT of HIV and syphilis from routine data is limited and the MTCT of HIV must be estimated from models. Estimates from the spectrum show an overall transmission of 12.6% of HIV up to the end of breastfeeding in Tanzania at the end of 2017 [19]. Community studies on the burden of HIV and syphilis among children are limited. Findings of community studies will complement the existing facility information on transmission rates and subgroups at higher risk, thus contributing to the fine-tuning of the interventions to achieve the elimination goal. This study aimed to describe the prevalence of HIV and syphilis among children aged 0–36 months and associated factors in Kilimanjaro region, northern Tanzania.

## Methods

### Study design and area

This was a community-based cross-sectional study conducted in Kilimanjaro region in north-eastern Tanzania. The study was part of the larger primary study that assessed neuro-developmental milestones/status of children aged 0–36 months and associated factors in all of the 7 districts of Kilimanjaro region [27]. The primary study included children from 0 to 36 months because this is a time of great cognitive, emotional, and social development. They also used 0–36-month-old children because the tool they used for the assessment of developmental milestones had been validated for children in that age group in other African settings.

Kilimanjaro region has seven districts, with an estimated population of 1.6 million people. Of those, one-third are women of reproductive age and approximately 150,000 children are below 3 years. The fertility rate (per woman) is one of the lowest at 4.2 compared with the national average of 5.2 children per woman. Most of the residents (up to 80%) engage in subsistence farming and live in rural areas [28].

### Study population

The study population included children aged 0 to 36 months of age and their mothers/primary caretakers, in seven districts of the Kilimanjaro region.

### Sampling method

From June 2010 to March 2011, a multistage sampling method was used to obtain a representative sample. The first stage was the creation of a sampling frame, which included the number of all children aged 0 to 36 months in each village/street of Kilimanjaro region, with a column for the cumulative population obtained from the National Bureau of Statistics [28]. The study needed to sample 50 clusters from the region. The second stage involved the selection of clusters. We divided the total population of children aged 0–36 in the region by the number of clusters (50) to get the sampling interval. A random cluster sampling method then conducted sampling, and 50 clusters were selected from seven districts proportionate to the population size. The first cluster was determined by multiplying a computer-generated random number between 0 and 1 and the sampling interval. By adding the previous number to the sampling interval, the subsequent clusters were located, to a total of 50 clusters. The third stage involved the selection of children from the 50 clusters. A compact segment sampling method was applied to select 50 children aged 0 to 36 months in each cluster. Segmented sampling involves the division of the selected clusters into segments of 50 children, and each segment was allocated a number, and one number was selected at random. In each selected segment, all the children meeting the inclusion criteria were enrolled. Recruitment was conducted by visiting all households in the segment until 50 children were enrolled. In the case that fewer than 50 eligible children were enrolled in a segment, enrolment went to a second randomly selected segment [27]. To maximize recruitment, prior information was given so the mothers/caretakers and their eligible children would be available on the day of the interview.

### Interviews

Face-to-face interviews were conducted using a questionnaire. Information collected included social, economic, and demographic characteristics, such as age, sex, maternal education level, marital status, occupation, income, household facilities, reproductive and child health, and breastfeeding history. Information on the use of health services during pregnancy of the child in the study, during delivery and during postnatal period, was also collected.

### Blood samples collection and laboratory methods

Dried blood spots (DBS) were prepared using Whatman filter papers on blood samples collected by finger or heel prick from all the children. Whatman filter papers were labelled with a corresponding participant identification number. The filter papers were air-dried and sealed in airtight plastic bags. The samples were sent to the Kilimanjaro Christian Medical Centre (KCMC) Clinical Laboratory, where they were stored in a minus 20-degree-Celsius freezer prior to analysis. Laboratory analysis was done using commercial tests, in accordance with the manufacturer’s instructions. HIV testing was done by detecting HIV specific IgG antibodies from eluted serum by enzyme-linked immunosorbent assay (ELISA) (Vironostika HIV Ag/Ab (bioMérieux)). All HIV ELISA positive samples of the children aged ≤ 18 months were subjected to a deoxyribonucleic acid polymerase chain reaction (PCR) using the Roche Amplicor HIV-1 DNA Test, v1.5. HIV was diagnosed by a positive result of PCR in children < 18 months and by positive ELISA test in children aged 18–36 months.

Syphilis-specific IgG antibodies were detected from eluted serum by human syphilis screening ELISA (Human Gesellschaft für Biochemical und Diagnostica mbH, Germany). All samples reactive for *Treponema pallidum* antibodies were retested and confirmed by the *Treponema pallidum* hemagglutination test (TPHA). Syphilis was diagnosed by positive results of both tests.

### Data analysis

Data obtained from interviews and laboratory test results were entered and verified for consistency. Statistical analysis was performed using the Statistical Package for Social Sciences (SPSS) for Windows version 20.0. HIV infection and syphilis infection status were assigned as the dependent variable, and all other epidemiologic variables were assigned as the independent variables. Descriptive statistics were used to summarize the data. Results are expressed as numbers and percentages. Differences between groups were compared using Fisher’s exact test or chi-squared test as appropriate. Bivariate and multivariable logistic analyses were used to assess factors associated with HIV and syphilis infection among children. Odds ratios (OR), along with their 95% confidence intervals, were calculated to measure the effect of exposures on outcomes (HIV and syphilis). A *p* value of < 0.05 was considered statistically significant. All the variables that were significant at 5% level in the bivariate analysis were included in the multivariable logistic regression model to get independent predictors of HIV infection.

## Results

### Background characteristics of the children

In this study, 2452 children were enrolled, 52% were male. Of these children, 95% of the informants were mothers. The mean age of the children was 12.4 months (SD = 7.8). Most of the children (71.6%) resided in rural areas. Of those who gave information on ANC and delivery care, a total of 96.5% reported to have undertaken HIV counselling and testing and 87% reported that the delivery of the child was in a health facility. Almost all the children (99.4%) had completed immunizations for their age. Other background characteristics are shown in Table 1.

**Table 1:** Background characteristics of 2452 children of 0–36 months of age in Kilimanjaro region, Tanzania

Characteristics	*N*	%
Child’s sex		
Male	1270	51.8
Female	1182	48.2
Child’s age groups in months		
0–6	699	28.5
7–12	648	26.4
13–18	506	20.7
> 18	599	24.4
District of residence		
Same	427	17.4
Mwanga	183	7.5
Rombo	511	20.8
Moshi District	645	26.3
Moshi Municipal	245	10.0
Hai	234	9.5
Siha	207	8.4
Residence type		
Rural	1755	71.6
Urban	697	28.4
Antenatal HIV testing (*N* = 2388)		
Yes	2305	96.5
No	83	3.5
Reported ANC HIV status (*N* = 2311)		
Positive	83	3.6
Negative	2228	96.4
Child’s delivery place (*N* = 2427)		
Health facility	2116	87.2
Home	311	12.8
Who assisted delivery (*N* = 2418)		
Trained provider	2121	87.7
Untrained person	297	12.3
Child’s birth weight (Kilograms) (*N* = 2375)		
Below 2.5	121	5.1
2.5 and above	2254	94.9
Immunization status for age (*N* = 2386)		
Complete	2371	99.4
Incomplete	15	0.6
Presence of child health card		
Yes	2302	93.9
No	150	6.1
Who gave information		
Mother	2329	95.0
Other	123	5.0

### Prevalence and factors associated with HIV infection

A total of 42 children were positive for HIV (ELISA test and/or PCR) giving an overall prevalence of HIV of 1.7% (95% CI, 1.3–2.3). Half of these children (*n* = 22; 52%) were less than 7 months. The prevalence of HIV was significantly higher among children aged 0–6 months (3.1%) compared with those aged 7–36 months (odds ratio, 2.82; 95% CI, 1.53–5.19) (Table 2).

**Table 2:** Background characteristics by HIV-seropositive status among the 2452 children in Kilimanjaro region

Variables	*N*	HIV positive, *n* (%)	*p* value	
All children	2452	42 (1.7)		
Child’s sex				
Male	1270	24 (1.9)	0.535	
Female	1182	18 (1.5)		
Child’s age groups (months)				
0–6	699	22 (3.1)	0.006	
7–12	648	9 (1.4)		
13–18	506	5 (1.0)		
> 18	599	6 (1.0)		
Child’s district				
Same	427	5 (1.2)	0.058	
Mwanga	183	5 (2.7)		
Rombo	511	14 (2.7)		
Moshi District	645	5 (0.8)		
Moshi municipal	245	6 (2.4)		
Hai	234	6 (2.6)		
Siha	207	1 (0.5)		
Child’s residence				
Rural	1755	34 (1.9)	0.174	
Urban	697	8 (1.1)		
Child’s caregiver (*N* = 2417)				
Both parents	1861	29 (1.6)	0.023	
Single parent	487	7 (1.4)		
Others	69	4 (5.8)		
Maternal antenatal care (*N* = 1967)				
Yes	1930	31 (1.6)	0.126	
No	37	2 (5.4)		
Maternal antenatal HIV testing (*N* = 2388)				
Yes	2305	36 (1.6)	0.002	
No	83	5 (6.0)		
Reported ANC HIV status of mother (*N* = 2311)				
Positive	83	10 (12.0)	< 0.001	
Negative	2228	26 (1.2)		
Child’s delivery place (*N* = 2427)				
Health facility	2116	37 (1.8)	0.223	
Home	311	2 (0.6)		
Who assisted delivery (*N* = 2418)				
Trained provider	2121	37 (1.7)	0.221	
Untrained person	297	2 (0.7)		
Breast feeding type (*N* = 1771)				
On demand	1705	24 (1.4)	0.001	
Time table	66	5 (7.6)		

The prevalence of HIV among children varied significantly by the child’s caretaker’s status, antenatal HIV testing and breastfeeding type. There was no significant difference in HIV infection by gender, district, residence, or the place/person conducting the child’s delivery (Table 2).

In bivariate analysis, children who were orphans had significantly 4 times greater odds of being HIV positive than those with single or both parents (Table 3). Women with negative HIV results during ANC had 89% lower odds of having a child with HIV than those who were HIV positive.

**Table 3:** Bivariate and multivariate analysis on factors associated with HIV among children aged 0–36 months in Kilimanjaro region

Variable	HIV positive, *n* (%)	Crude odds ratio	Adjusted odds ratio
Child’s age categories (months)			
0–6	22 (3.1)	1	1
7–12	9 (1.4)	0.43 (0.2, 0.95)	0.48 (0.7, 4.6)
13–18	5 (1.0)	0.31 (0.12, 0.82)	0.75 (0.7, 6.4)
> 18	6 (1.0)	0.31 (0.13, 0.77)	0.83(0.8, 11.0)
Child’s caretaker			
Both parents	29 (1.6)	1	
Single parent	7 (1.5)	0.92 (0.40, 2.12)	–
Others	4 (6.2)	4.08 (1.39, 12.0)	–
Antenatal HIV testing			
Yes	36 (1.6)	1	
No	5 (6.0)	4.04 (1.54, 10.6)	3.01(0.91–11.7)
Antenatal HIV results			
Positive	10 (10.0)	1	1
Negative	26 (1.2)	0.11 (0.05, 0.23)	0.2 (0.1, 0.4)
Breast feeding type			
Time table	5 (7.6)	1	1
On demand	24 (1.4)	0.17 (0.06, 0.47)	0.14 (0.1, 0.4)

In multivariate analysis, a negative antenatal HIV test of the mother (AOR, 0.2; 95% CI, 0.1, 0.4), and breastfeeding on demand (AOR, 0.14; 95% CI, 0.1, 0.4) remained significantly associated with child HIV status (Table 3).

### Prevalence and factors associated with syphilis

A total of 10 children had syphilis, giving an overall prevalence of 0.4% (10/2452). Two children with syphilis were ≤ 6 months (Table 4). All of the ten syphilis-positive children were from rural residences in Moshi rural district. Significantly more syphilis-positive children were from mothers consuming alcoholic compared with non-drinkers (OR, 4.94; 95% CI, 1.28, 19.2). Syphilis infection did not differ in other socio-demographic variables (Table 4). Given that only one variable was significantly associated with syphilis, multivariable logistic regression analysis was not performed.

**Table 4:** Distribution of syphilis by demographic characteristics in children of 0–36 months in Kilimanjaro region, *N* = 2452

Variable name	*N*	Syphilis positive, *n* (%)	Crude OR (95% CI)	*p* value
Child’s sex				
Female	1182	2 (0.2)	1.0	
Male	1270	8 (0.6)	3.74 (0.79–17.6)	0.111
Child’s age categories (months)				
0–6	699	2 (0.3)	1.0	
7–12	648	3 (0.5)	1.62 (0.27–9.73)	
13–18	506	2 (0.4)	1.38 (0.19–9.85)	
> 18	599	3 (0.5)	1.75 (0.29–10.5)	0.933^†^
Child’s residence				
Rural	1755	10 (0.6)	–	–
Urban	697	0 (0)	–	–
Child’s caretaker (*N* = 2417)				
Both parents	1861	8 (0.4)	1.0	
Single parent/others	506	2 (0.3)	1.19 (0.25–5.62)	0.99
Maternal antenatal care (*N* = 1967)				
Yes	1930	9 (0.5)	1.0	
No	37	1 (2.7)	5.93 (0.73–48.0)	0.173
Presence of RCH card				
Yes	2302	9 (0.4)	1.0	
No	150	1 (0.7)	1.71 (0.22–13.6)	0.469
Mother drinking alcohol (*N* = 2368)				
No	1605	3 (0.2)	1.0	
Yes	763	7 (0.9)	4.94 (1.28–19.2)	0.016

*RCH*, reproductive and child health; ^†^*p* for trend

## Discussion

This community-based study found a low prevalence of both syphilis (0.4%) and HIV (1.7%) infection among children aged (0–36 months) in Kilimanjaro region, Tanzania. Predictors of HIV infection among children included breastfeeding type, antenatal HIV testing, and antenatal HIV positive results. There was no child with HIV-syphilis co-infection. The population-based estimate of HIV among children is lower than the HIV prevalence observed in EID samples collected at 6–8 weeks in Tanzania of 4.8% in 2017 and estimates form spectrum shows an overall transmission of 12.6% up to the end of breastfeeding (19).

In this study, syphilis was only found among children from rural areas, and among mothers consuming alcohol. This may be due to the fact that women in rural areas, where they are mostly served by dispensaries, have less access to preventive health care services, especially screening for syphilis during pregnancy. The Tanzanian Service Provision Assessment (SPA) of 2014 showed that rural clinics reported a greater lack of equipment and stock-outs than urban facilities [29]. Several studies have shown that it is common for ANCs in low-income countries to lack the tools needed for appropriate syphilis screening, which could lead to missed opportunities for timely treatment and prevention of congenital syphilis [10, 13, 17, 22, 26]. A study conducted in Moshi urban by Katanga et al. [13] showed that syphilis screening is not given a priority as 89.4% of pregnant women reported not being tested for syphilis, compared with only 1% who reported they were not tested for HIV. Providers and policy makers should be reminded that antenatal syphilis screening is cost effective even in programs where antenatal syphilis prevalence is below 0.1% [10, 22]. As the goal is dual elimination of both HIV and congenital syphilis among children by 2020, the need for strategies that can improve syphilis testing during pregnancy, prompt treatment and checking for congenital syphilis cannot be overemphasized [10, 25].

The overall HIV prevalence of 1.7% among children is in line with the low HIV prevalence in Kilimanjaro region of 2.6% according to the 2016–2017 Tanzania HIV Impact Survey [30]. The low HIV prevalence observed among children in the community may be attributed to the ongoing national prevention of mother-to-child transmission of HIV (PMTCT) program that started in 2004 in Tanzania. The PMTCT program is performing well in Tanzania and by the end of 2018 the testing rate was 99% (pregnant women who are tested for HIV) and an antiretroviral (ART) treatment coverage of 87% [15, 19, 21]. Even though this study was conducted in 2011, both ANC attendance and HIV testing rates were high at 98% and 97% respectively [19]. Though not significant, observation of a higher occurrence of HIV among 0–6-month-old children than that of older age groups needs to be investigated further. It may indicate/suggest that HIV-positive children who are not diagnosed early and therefore do not get into early care for lifelong ART end with mortality and therefore do not appear in older age groups.

Of concern is that a greater proportion of children with HIV infection were from women who reported that they were HIV negative during their first ANC visit when tested. Some were from women who were tested, knew their status but did not test the children. HIV early infant diagnosis (EID) does not have high coverage like testing rates among pregnant women. A study that was conducted in 2015–2016 in north-eastern Tanzania showed that only 57.1% of the HIV-exposed children accessed received an EID [20]. At the end of 2018, the EID testing rate was 55% in Tanzania, showing that we are missing the opportunity to diagnose and link children into care in a timely manner [19]. Further, the national guidelines stipulate that all HIV-negative pregnant women at first booking should be retested for HIV in the third trimester. Retesting in the third trimester is very low in Tanzania (< 20%) implying incident infections during late pregnancy are missed and thus increasing the risk of HIV infection in children [19]. A study in Mwanza showed sero-conversion during pregnancy was 2% on repeat testing for previously HIV-negative antenatal mothers [31]. This finding further highlights the importance of strengthening interventions for repeated testing for HIV-negative mothers during the antenatal period in the 3rd trimester or during labor and delivery.

HIV infection was associated with having a caretaker who is not a parent and breastfeeding on time table. This suggests that these children might have been orphans cared for by relatives who are dependent on an alternative source of milk for feeding the children [18]. Studies have consistently shown that ART reduces mortality among HIV-infected individuals, reduces morbidity, and improves quality of life [9]. This means that in order to achieve the 90-90-90 target by 2020, the HIV program in Tanzania will need to increase the proportion of HIV-infected persons who are on antiretroviral treatment as well as achieving viral suppression. About 68% of adults estimated to be LWHIV are on ART treatment and the prevalence of viral load (VL) suppression ranges from 32.4 to 66.8% in Tanzania [9, 30].

This study identified existing opportunities for the elimination of HIV and syphilis in Kilimanjaro region, including low HIV and syphilis prevalence among adults, low prevalence of HIV/syphilis among children aged 0–36 months, and high awareness on PMTCT of HIV and syphilis, coupled with high antenatal and postnatal attendance. Identified barriers for HIV/syphilis elimination include the missed opportunity through low retesting of HIV at 3rd trimester, low priority or lack of syphilis testing during ANC visits, and the missed opportunity to offer early infant diagnosis for exposed children.

The strength of this study is the large sample size, drawn from the entire seven districts in the region. Interviewing caregivers and examining children at their homes allowed objective assessment of socio-demographic and household conditions. The children’s health status, physical abilities, and cognitive function were better assessed in the home environment. Community testing shows a picture of what is going on more precisely than facility-based studies [32].

A weakness of this study is its cross-sectional design. Mothers who experienced abortions, stillbirths, perinatal, infancy, and early deaths of children, who are likely to have HIV or syphilis infection, were not captured in this study since the study recruited mothers with live children aged 0–36 months. The HIV and syphilis transmission observed may therefore be an underestimation of the true burden. Affected children in orphanages and special schools, admitted, or attending hospital were also not accessed by this study. Another weakness might have been an information bias. This may have occurred in the information on service use during pregnancy and during the postpartum period for those other caregivers and not the mothers of the children provided whose cards were not found or the information.

Generalization of these results to other regions may be limited given a marked regional variation of HIV prevalence among adults aged 15–49 years, ranging from < 1% in Lindi and Zanzibar to 11% at Njombe and Iringa, while that of Kilimanjaro is 2.6% [30]. Likewise, the prevalence of syphilis among pregnant women shows marked regional variation with 0.7 to 1% in Kilimanjaro compared with 7% in other regions like Mwanza [7, 8, 13]. These variations emphasize the need to have regional specific data that will guide interventions.

## Conclusions and recommendations

This study aimed to investigate the prevalence and predictors of HIV and syphilis among children and found that the prevalence of mother-to-child transmission of HIV and syphilis infections was low in Kilimanjaro region. There are several opportunities to achieve dual elimination of MTCT for HIV and syphilis, such as strengthening syphilis screening during pregnancy, enhancing HIV retesting in the 3rd trimester or at labor, and the integrated reproductive and child health programs where exposed children can be tested early and started into care. There is a need for focused awareness campaigns on elimination goals for the two infections, a need to raise awareness of syphilis and its consequences, and the importance of early testing and timely treatment.

## Data Availability

The datasets used and/or analyzed during the current study are available from the corresponding author on reasonable request.

## Abbreviations

AIDS: Acquired immunodeficiency syndrome
ANC: Antenatal care
DBS: Dried blood spot
DNA: Deoxyribonucleic acid
ELISA: Enzyme-linked immunosorbent assay
LWHIV: Living with HIV
MTCT: Mother-to-child transmission
PCR: Polymerase chain reaction
PMTCT: Prevention of mother-to-child transmission
RCH: Reproductive and child health
WHO: World Health Organization
